# Functional Benefits of Fermented Sesame Oilcake in the Diet of Nile Tilapia, *Oreochromis niloticus*: Insights Into Immunity, Oxidative Status, and Gut Microbiota

**DOI:** 10.1155/anu/4050656

**Published:** 2026-07-16

**Authors:** Seyyed Morteza Hoseini, Morteza Yousefi, Alireza Afzali-Kordmahalleh, Seyed Hossein Hoseinifar, Hamed Abdollahpour, Mert Minaz

**Affiliations:** ^1^ Inland Waters Aquatics Resources Research Center, Iranian Fisheries Sciences Research Institute, Agricultural Research, Education and Extension Organization, Gorgan 4916687631, Golestan, Iran, ifsri.ir; ^2^ Department of Veterinary Medicine, RUDN University, Miklukho-Maklaya Street, Moscow 117198, Russia, rudn.ru; ^3^ Department of Aquatic Animal Health, Faculty of Veterinary Medicine, University of Tehran, Tehran 1419963114, Iran, ut.ac.ir; ^4^ Department of Fisheries, Gorgan University of Agricultural Sciences and Natural Resources, Gorgan, Iran, gau.ac.ir; ^5^ Comparative Endocrinology and Integrative Biology, Centro de Ciências do Mar, Universidade do Algarve, Faro, Portugal, ualg.pt; ^6^ Department of Aquaculture, Faculty of Fisheries, Recep Tayyip Erdoğan University, Rize, Türkiye, erdogan.edu.tr

**Keywords:** antioxidant defense, fermentation, functional feed, gut microbiota, immune response

## Abstract

This study evaluated the effects of dietary inclusion of fermented sesame oilcake (FSOC) on growth performance, antioxidant responses, immune parameters, bactericidal activity, and gut microbial composition in juvenile Nile tilapia, *Oreochromis niloticus*. FSOC was produced by aerobic solid‐state fermentation using *Saccharomyces cerevisiae*. Fish (5‐month‐old, 13.31 ± 0.73 g, all‐male) were randomly distributed into three treatment groups and fed diets containing 0% (control [CTL]), 10% (S‐10), or 20% (S‐20) FSOC for 8 weeks. Growth performance, feed conversion ratio, and survival were not significantly affected by FSOC inclusion (*p* > 0.05). However, dietary FSOC significantly enhanced several immunological parameters, including lysozyme activity and total immunoglobulin (Ig) levels in plasma, liver, kidney, and skin mucus (*p* < 0.05). Antioxidant markers such as superoxide dismutase (SOD), catalase (CAT), glutathione peroxidase (GPx), and reduced glutathione (GSH) were significantly elevated in hepatic and intestinal tissues of FSOC‐fed fish, accompanied by a significant reduction in malondialdehyde (MDA) levels (*p* < 0.05), indicating improved oxidative status. Skin mucus from FSOC‐fed tilapia exhibited significantly greater bactericidal activity against *Aeromonas hydrophila* and *Streptococcus iniae*. Gut gene expressions showed different patterns in the S‐10 and S‐20 treatments, as the S‐10 treatment showed only an upregulation in tumor necrosis factor‐a (*tnf-α*) expression, while the S‐20 treatment showed upregulations in various anti‐inflammatory, proinflammatory, antioxidant, and chaperon gene expressions. Additionally, gut microbiota analysis revealed that FSOC supplementation significantly increased the abundance of beneficial microbes (*Saccharomyces* sp., *Lactobacillus* sp., and *Bacillus* sp.) while reducing the presence of opportunistic pathogens (*Aeromonas* sp., *Vibrio* sp., and *Streptococcus* sp.). Overall, these findings suggest that FSOC is a promising functional feed ingredient capable of improving immune competence, oxidative resilience, and gut health in Nile tilapia without compromising growth.

## 1. Introduction

Reducing the environmental footprint of aquaculture increasingly relies on nutrient upcycling strategies that valorize regionally abundant agro‐industrial residues [1]. Within this context, oilseed cakes constitute a significant yet underutilized biomass resource; despite their large‐scale production, many are relegated to low‐value applications [2, 3]. As global aquafeed production expands, the need to replace conventional protein sources, such as fishmeal and soybean meal, with sustainable alternatives becomes increasingly urgent [4]. Sesame oilcake emerges as a particularly promising candidate due to its high availability in warm‐climate agricultural systems—such as India, Sudan, Myanmar, China, and Tanzania [5]—and a nutritional profile comparable to soybean [6]. While sesame oilcake has demonstrated potential for incorporation into aquafeeds across diverse species [7–10], its broader application is constrained by antinutritional factors (ANFs), a common challenge shared with other low‐cost plant proteins [11, 12]. To successfully incorporate these alternative ingredients within circular bioeconomic frameworks, fermentation technologies offer transformative potential. Fermentation improves digestibility and reduces ANFs, thereby upgrading the nutritional quality of oilseed by‐products and facilitating their use as functional aquafeed ingredients [13]. Consequently, existing literature indicates that fermented sesame oilcake (FSOC) can be incorporated into fish diets at higher inclusion levels compared to non‐FSOC in Nile tilapia, *Oreochromis niloticus*, and ruho carp, *Labeo rohita* [12, 14].

Beyond basic nutritional provision, evaluating the efficacy of functional ingredients requires an understanding of their impact on gastrointestinal health. The fish gut is a multifunctional organ critically involved in nutrient digestion, absorption, and immunity [15]. This organ harbors a complex microbial community whose structure influences various physiological pathways, affecting not only localized gut‐dependent processes but also systemic host functions [16, 17]. Emerging research suggests that dietary interventions, including the application of fermented feeds, can beneficially alter gut microbial communities and influence host immune and oxidative pathways [18]. While previous works have evaluated fermented ingredients in various aquaculture species (review by Siddiqui, et al. [19]), few studies have linked these microbiome shifts with the expression of genes related to immunity, stress response, and oxidative regulation [20, 21]. Crucially, such integrated molecular and microbiological data are currently unavailable for fish fed FSOC, highlighting a significant knowledge gap.

Considering the above, the present study aimed to assess the effects of dietary FSOC in Nile tilapia by monitoring fish growth performance and diverse biological responses, particularly gut gene expression and the abundance of key microbial taxa. Nile tilapia was selected as the experimental model because it is one of the most important farmed fish species worldwide, is highly adaptable to plant‐based diets, and serves as a widely established model for nutritional, immunological, and gut‐health studies. Its omnivorous feeding habit and robust physiological responses make it an ideal candidate for evaluating functional feed ingredients derived from upcycled plant by‐products [22, 23].

## 2. Materials and Methods

### 2.1. Solid‐State Fermentation

Fermentation was performed according to Hassaan et al. [24]. Active dry baker’s yeast (*Saccharomyces cerevisiae*; Saf‐Levure, Saf‐Levure Co., Maisons‐Alfort Cedex, France; declared viable count 8 × 10^9^ cell/g) served as the fermentative inoculum. Immediately before use, 100 mg of yeast was suspended in 100 mL sterile distilled water, vortexed, and allowed to hydrate for 15 min at an ambient temperature. This primary suspension was serially diluted 1:100 (v/v) in sterile distilled water to yield the working inoculum. For each kilogram of milled sesame oilcake (as‐fed basis), 12 mL of the working yeast inoculum and 550 mL additional sterile distilled water were added while mixing to achieve a moist crumb‐like substrate. The hydrated substrate was distributed in shallow trays, covered loosely with perforated aluminum foil to limit desiccation while permitting gas exchange, and incubated at 40°C for 48 h in a temperature‐controlled chamber. Fermentation progress was monitored visually (mycelial/yeast growth and aroma development). At the end of incubation, the fermented material (FSOC) exhibited a cohesive, creamy texture and a characteristic fermentation odor. The fermented substrate was dried in a forced‐air oven at 60°C for 72 h, cooled to room temperature under desiccation‐protected conditions, and remilled (electric mill; <500 µm sieve). The finished FSOC was sealed in moisture‐impermeable bags, flushed with air, and held at 4°C pending diet manufacture.

### 2.2. Preparation of Experimental Diets

Experimental diets were formulated using the WUFFDA least‐cost formulation software (University of Georgia, USA) to meet or exceed the known nutrient requirements for juvenile Nile tilapia, *O. niloticus*, while incorporating graded levels of FSOC. Three experimental diets were prepared: a control (CTL) diet without FSOC, a diet containing 100 g FSOC per kg of diet (S‐10; ~10% inclusion), and a diet containing 200 g FSOC per kg of diet (S‐20; ~20% inclusion). These levels were chosen to allow for formulating cost‐effective diets without the need to add expensive dense protein sources to diets. The ingredient composition and calculated nutrient profiles of all diets are presented in Table 1. All dry ingredients were first sieved through a 500 µm mesh, weighed accurately, and blended in a horizontal ribbon mixer for 10 min to ensure homogeneity. To achieve pelleting consistency, warm water was gradually added at a ratio of 400 g/kg (~40% v/w) during mixing. The resulting moist mash was then cold‐extruded using a meat grinder fitted with a 3 mm die, cut into pellets ~3 mm in length, and dried using forced ambient air at or below 30°C until the moisture content dropped below 10%. The dried pellets were gently crumbled when necessary to match the mouth size of juvenile tilapia, sealed in airtight polyethylene bags, and stored at 4°C until use. All experimental diets were produced in a single batch to eliminate interbatch variation and ensure consistency across feeding trials.

**Table 1:** Formulation and proximate composition of experimental diets containing 0% (CTL), 10% (S‐10), and 20% (S‐20) fermented sesame oilcake (FSOC) for juvenile Nile tilapia.

Ingredients (g/kg)	CTL	S‐10	S‐20
Soybean meal^a^	450	400	350
Wheat meal	260	260	260
Poultry slaughterhouse by‐product^b^	250	200	150
Fermented sesame oilcake^c^	0	100	200
Canola oil	15	15	15
Mineral premix^d^	5	5	5
Vitamin premix^d^	5	5	5
Lysine^e^	0	1	2
Methionine^e^	5	4	3
Sodium chloride	10	10	10
Proximate composition (g/kg)			
Moisture	102.5	94.0	85.5
Crude protein	375.5	378.5	381.5
Crude fat	84.5	86.7	87.9
Crude fiber	38.8	42.2	45.5
Crude ash	65.2	66.8	67.6

*Note:* Ingredient levels are presented in g/kg of diet.

^a^Crude protein: 431 g/kg.

^b^Crude protein 536 g/kg and crude fat 198 g/kg.

^c^Crude protein 515 g/kg, crude fat 143 g/kg, crude fiber 58 g/kg, and crude ash 94 g/kg.

^d^As reported by Yousefi et al. [25].

^e^Evonik Degusa; 99% purity.

### 2.3. Experimental Fish and Acclimation

Juvenile Nile tilapia (all‐male Iranian population, 5‐month‐old) with an initial mean body mass of 13.31 ± 0.73 g (mean ± SD) were obtained from a local hatchery and transported in aerated tanks to the Aquatic Animal Nutrition Laboratory, University of Tehran. Upon arrival, fish were acclimated for 14 days in 200 L polyethylene holding tanks supplied with filtered, aerated water and fed the CTL basal diet at 2% of body biomass per day, administered twice daily. Health status was assessed visually throughout acclimation, and only clinically normal individuals without external lesions were selected for the feeding trial. A completely randomized design was implemented, comprising three dietary treatments (CTL, S‐10, and S‐20) with three replicate tanks per treatment. The sample size in this experiment was according to previous studies [26]. Experimental units consisted of 200 L tanks (working volume 125 L) integrated into a recirculating aquaculture system equipped with mechanical filtration (wool/floss cartridges), biological filtration using >30 mm pumice media, and ultraviolet disinfection. Continuous aeration was maintained using air stones connected to a central blower. Fourteen fish were stocked per tank at an initial stocking density of ~1.5 kg/m^3^ (exact weight per tank to be confirmed). Simple randomization was applied for the tanks and fish allocations. Fish were hand‐fed their assigned diets twice daily at 08:00 and 16:00, starting at a ratio of 30 g/kg biomass per day (equivalent to 3% body weight), with feeding rates adjusted every 14 days based on the tank biomasses. The feeding trial lasted for 8 weeks, after which, the fish growth performance was calculated as follows:

Specific growth rate SGR;%/d=100×ln final weight g−ln initial weightg56,

Weight gain %=100×Final weight g−Initial weight gInitial weight g,

Feed conversion ratio FCR=Feed intake gWeight gain g,

Survival %=100×Final number of fishInitial number of fish.

Water quality parameters, including temperature, dissolved oxygen, and pH, were monitored daily using a calibrated portable multiparameter meter, while total ammonia nitrogen was assessed three times weekly and unionized ammonia (NH_3_─N) was estimated based on pH and temperature. Mean (±SD) water quality values throughout the study were temperature 26.5 ± 0.5°C, pH 7.30 ± 0.36, dissolved oxygen 5.87 ± 0.42 mg/L, and unionized ammonia 0.010 ± 0.001 mg/L. All experimental procedures were conducted following the Institutional Animal Care and Use Committee (IACUC) guidelines of the University of Tehran under Approval Code IR.UT.VETMED.REC.1403.063.

### 2.4. Sample Collection and Processing

At the end of the experiment, three fish were randomly (simple randomization) selected from each tank and anesthetized in a clove extract solution (3 g/L). Blood samples were collected from the caudal vein using heparinized syringes, after which the fish were euthanized by spinal cord severance. Tissue samples, including hindgut, liver, and kidney, were immediately excised and snap‐frozen in liquid nitrogen. Blood plasma was separated by centrifugation at 2800 × g for 10 min at 4°C. Equal volumes of plasma from each fish within a tank were pooled to obtain a sufficient sample for subsequent analyses (*n* = 3 per treatment). The pooled plasma samples were stored at −70°C until use. A portion of the hindgut was snap‐frozen in liquid nitrogen and transferred to a −70°C freezer for molecular analyses. The remaining hindgut, liver, and kidney tissues were homogenized in phosphate buffer (pH 7.0) at a 1:1 ratio and centrifuged at 18,928 × g for 15 min at 4°C. The resulting supernatants were collected and stored at −70°C until further biochemical analyses (*n* = 3 per treatment). To minimize ex vivo oxidation of reduced glutathione (GSH), all tissue homogenization procedures were conducted using ice‐cold buffers, samples were maintained at 4°C throughout processing, and supernatants were rapidly separated and stored at −70°C until biochemical analyses.

Another three fish per tank were caught, anesthetized as described above, and used for skin mucus collection. The anesthetized fish were rinsed gently with sterile isotonic saline (0.85% NaCl) to remove the loosely adherent material. The mucus was then collected by gentle scraping along the lateral surface using a sterile plastic spatula. Samples from three fish per tank were pooled to have one sample per tank. The pooled samples were mixed with cold phosphate‐buffered saline (PBS; 50 mM, pH 7.0) at a 1:5 (w/v) ratio. Mucus suspensions were vortexed and clarified by centrifugation (5000 × g, 10 min, 4 °C), and supernatants were kept at −70°C for further analysis.

### 2.5. Analysis

All analyses in this experiment were blindly conducted to minimize potential confounders such as the order of treatments and measurements or animal/tank locations.

#### 2.5.1. Analysis of Plasma Immunological Parameters

Lysozyme activity was determined using a turbidimetric assay [27] against *Micrococcus luteus* suspended in 50 mM phosphate buffer (pH 6.2) adjusted to an optical density (OD) of 0.5–0.6 at 530 nm. Twenty‐five microliters of plasma was added to 975 µL of the bacterial suspension, and the decrease in OD was recorded at 25°C over 5 min at 1‐min intervals using a spectrophotometer. One unit (U) of lysozyme activity was defined as a 0.001 decrease in OD per minute, and results were expressed as U/mL of plasma.

Alternative complement (AC) pathway activity was assessed following a modified hemolytic assay protocol [28]. Serial dilutions of plasma (ranging from 0.313% to 10% v/v) were prepared in magnesium–EGTA barbital buffer containing gelatin. Twenty‐five microliters of each dilution was mixed with 50 µL of a 3.33% sheep erythrocyte suspension in the same buffer and incubated for 90 min at room temperature with gentle agitation. Reactions were terminated by the addition of EDTA to a final concentration of 9 mM. Samples were then centrifuged at 5000 × g for 10 min, and the degree of hemolysis was quantified by measuring the absorbance of the supernatant at 412 nm. AC values, representing the plasma volume required to cause 50% hemolysis, were calculated via linear interpolation.

Total immunoglobulin (Ig) concentration was measured by polyethylene glycol (PEG) precipitation. Plasma samples were mixed 1:1 (v/v) with 12% PEG prepared in PBS and incubated for 2 h at room temperature with gentle shaking. Following centrifugation at 7000 × g for 15 min, the soluble protein content of both PEG‐treated and untreated supernatants was measured. Total Ig concentration was calculated as the difference in protein content between the untreated and PEG‐precipitated samples and expressed as mg/mL of plasma, following Siwicki and Anderson [29].

#### 2.5.2. Determination of Immunological Parameters in Skin Mucus, Liver, and Kidney

Lysozyme activity in the skin mucus, liver, and kidney tissues was measured using the same turbidimetric method described for plasma samples, with slight modifications in the sample volume. Specifically, volumes of 50 μL, 15 μL, and 15 μL were used for the skin mucus, liver, and kidney extracts, respectively. The specific activity of lysozyme in each tissue was calculated by normalizing enzyme activity to the corresponding soluble protein concentration in the extract. Total Ig levels in the skin mucus, liver, and kidney were also assessed using the same PEG precipitation method described for plasma, with appropriate volume adjustments. Total Ig levels were expressed as a percentage of the total soluble protein content in each tissue extract. Skin mucus alkaline phosphatase (ALP) activity was measured using a commercially available diagnostic kit (Zist Chem Co., Tehran, Iran) following the manufacturer’s instructions. Protease activity in the skin mucus was determined using azocasein as the substrate, according to the method described by Iversen and Jørgensen [30]. Both ALP and protease activities were normalized to the soluble protein concentration in the mucus extracts and expressed as specific activity (units per mg of protein).

#### 2.5.3. Determination of Skin Mucus Bactericidal Activity

The bactericidal activity of skin mucus samples was evaluated by measuring their ability to inhibit the growth of two fish pathogens, *Aeromonas hydrophila* and *Streptococcus iniae*, according to Hoseini et al. [31]. Both bacterial strains were obtained from the Iranian Research Organization for Science and Technology (Tehran, Iran). *A. hydrophila* (PTCC 1890) was cultured on standard nutrient agar, while *S. iniae* (PTCC 1887) was cultured on nutrient agar supplemented with 5% (v/v) defibrinated sheep blood to support its growth. Bacterial suspensions were prepared by harvesting colonies into a sterile 0.85% NaCl solution and adjusting the OD to 0.05–0.10 at 600 nm using a spectrophotometer to ensure standardized inoculum concentrations. Equal volumes (200 µL) of each bacterial suspension and skin mucus sample were mixed and incubated at room temperature (~25°C) for 1 h to allow for interaction between mucus antimicrobial factors and bacterial cells. Following incubation, the mixtures were serially diluted (10^−6^ to 10^−1^) in sterile saline, and 100 µL of each dilution was spread onto the respective culture media. Plates were incubated at 28°C for 24–48 h, after which colony‐forming units (CFUs) were enumerated. The bactericidal activity of the mucus was calculated as the percentage reduction in the CFU count relative to a CTL group, in which sterile 0.85% NaCl solution was used instead of mucus. The results were expressed as a percentage CFU inhibition, reflecting the antimicrobial potential of the mucus against each pathogen.

#### 2.5.4. Determination of Hepatic and Intestinal Antioxidant Parameters

Antioxidant enzyme activities and oxidative stress biomarkers were assessed in tissue homogenates using standard spectrophotometric methods. Superoxide dismutase (SOD) activity was measured based on its ability to inhibit the autoxidation of pyrogallol, following the method described by Marklund [32]. One unit of SOD activity was defined as the amount of enzyme required to inhibit the rate of pyrogallol autoxidation by 50%, with results expressed as U/mg of protein. Catalase (CAT) activity was determined according to the method of Goth [33], which quantifies the decomposition of hydrogen peroxide (H_2_O_2_) by the sample over a 60‐s interval. The rate of decrease in the H_2_O_2_ concentration was used to calculate CAT activity, expressed as U/mg protein. GSH peroxidase (GPx) activity was assessed based on the reduction of hydrogen peroxide using reduced GSH as a substrate, monitored through the development of a yellow–colored complex with Ellman’s reagent (5,5′‐dithiobis (2‐nitrobenzoic acid); DTNB), as described by Hu [34]. One unit of GPx activity was defined as the amount of the enzyme that oxidizes two molecules of GSH per minute. GSH reductase (Gr) activity was also determined using Ellman’s reagent, where the consumption of NADPH during the reduction of oxidized GSH was measured spectrophotometrically [34]. One unit of Gr activity corresponded to the oxidation of one micromole of NADPH per minute under assay conditions. GSH levels were quantified using Ellman’s reagent, which forms a yellow‐colored product upon reaction with free sulfhydryl groups. The absorbance was directly proportional to the GSH concentration in the sample, and results were expressed as µmol/g tissue [34]. Lipid peroxidation was estimated by determining malondialdehyde (MDA) concentrations, a terminal product of polyunsaturated fatty acid oxidation. MDA levels were measured by reacting to the sample with thiobarbituric acid (TBA) and heating the mixture at 95°C for 30 min, forming a pink chromogen. The absorbance of the MDA–TBA complex was measured spectrophotometrically, with higher absorbance indicating greater lipid peroxidation, as described by Buege and Aust [35].

#### 2.5.5. Determination of Intestinal Transcript Expression

The mRNA expression levels of key stress‐ and immune‐related genes including heat shock protein 70 (*hsp-70*), tumor necrosis factor‐alpha (*tnf-α*), interleukin‐1 beta (*il-1*β), interleukin‐6 (*il-6*), interleukin‐8 (*il-8*), interleukin‐10 (*il-10*), transforming growth factor‐beta (*tgf-β*), Kelch‐like ECH‐associated protein 1 (*keap1*), and nuclear factor erythroid 2‐related factor 2 (*nrf2*) were quantified in intestinal tissue samples using quantitative real‐time PCR (qRT‐PCR) with gene‐specific primers detailed in Table 2. Relative gene expression levels were normalized to the housekeeping gene *beta-actin* to CTL for variations in RNA quantity and quality. *Beta-actin* was used as the housekeeping gene as it showed narrow variation across the treatments (coefficient of variation = 2.13%). The comparative cycle threshold (Ct) method (2^−ΔΔCt^) described by Livak and Schmittgen [36] was employed to calculate fold changes in gene expression relative to the CTL group. Comprehensive protocols for RNA extraction, complementary DNA (cDNA) synthesis, primer validation, and qRT‐PCR conditions followed established procedures described in a previous study [37]. Total RNA was isolated from intestinal tissue samples using a commercial kit supplied by Denazist Co. (Tehran, Iran), and its integrity and purity were evaluated spectrophotometrically (A260/A280 ratio). To eliminate potential genomic DNA contamination, the purified RNA was subsequently treated with DNase I (Thermo Fisher Scientific Co., Waltham, MA, USA) and then reverse‐transcribed into cDNA using a kit from SMOBIO Technology Co. (Hsinchu City, Taiwan). qRT‐PCR was performed to assess gene expression, employing gene‐specific primers detailed in Table 2. Quantitative PCR reactions were conducted in a final volume of 25 µL under the following cycling conditions: initial denaturation at 95°C for 15 min, followed by 40 cycles of denaturation at 94°C for 30 s, annealing at primer‐specific temperatures for 40 s, and extension at 72°C for 30 s. Amplification and detection were carried out on an Applied Biosystems instrument (Waltham, MA, USA) using a SYBR Green master mix from Ampliqon A/S Co. (Odense M, Denmark), with Ct values recorded for each reaction. Relative expression levels of target transcripts were determined using the ΔΔCt method.

**Table 2:** Specific primer sequences used for quantitative real‐time PCR analysis of intestinal gene expression in juvenile Nile tilapia.

Gene name	Sequence		Amplicon size (bp)	Efficiency (%)	Accession number
*tnf-α*	F	GTCGTCGTGGCTCTTTGTTTA	113	96.4	AY428948.1
	R	GTGTTCTTCGCCTTTAGTGCT			
*il-1β*	F	AGGAAAACCAGCCCGTTGAA	92	93.6	XM_005457887.3
	R	TGCACGGGTCCTACAACATC			
*il-6*	F	GCAGGTGACTTCTCAGGTGA	124	94.6	XM_019350387
	R	GGAAATGGTGCTCAAACGCT			
*il-10*	F	ACGACAAGGGGGATGACTTC	100	92.7	XM_019358799.2
	R	TGAGCCTGATGAATAGGCCC			
*il-8*	F	AGCGCTTCAGGCTTCATCTA	126	98.1	NM_001279704.1
	R	CCAAAAGCACCACGATGGAG			
*tgf-β*	F	CAGCTTAAGTTTGTGGCGCT	98	97.3	XM_005463992
	R	TGTCACACTTCAGCGCTTTG			
*hsp-70*	F	TCAAACGCAACACCACCATC	145	96.1	XM_019357557.2
	R	GTCAGCTCAAACTTGCCCAG			
*keap1*	F	AATCGGAGTCGGAGTCATCG	143	97.9	XM_003447926.4
	R	CACCGATACGCCGTGTTAAC			
*nrf2*	F	TCGCAATTTCCTCTCACCCA	100	94.7	XM_025901593.1
	R	AGACACGCATTGGTTCTCCT			
*Beta-actin*	F	ATGGTGGGTATGGGTCAGAAA	139	95.6	XM_003443127.5
	R	AGGTGTGATGCCAGATCTTCT			

*Note*: The table includes gene targets, forward (F) and reverse (R) primer sequences, amplicon sizes (bp), and corresponding GenBank accession numbers.

#### 2.5.6. Determination of Specific Intestinal *Microb*es

The relative abundances of key bacterial genera, including *Saccharomyces* sp., *Lactobacillus* sp., *Bacillus* sp., *Aeromonas* sp., *Streptococcus* sp., and *Vibrio* sp., within the intestinal microbiota of the fish were quantified using molecular techniques. Species‐ or genus‐specific primers were designed and validated for each target microorganism (see Table 3). Primer specificity for each bacterial target was confirmed by melting curve analysis and agarose gel electrophoresis. Quantification was performed via qRT‐PCR, and the frequency of each microbe was expressed as a proportion of the total bacterial population, determined using universal 16S rRNA gene primers. This relative quantification approach allowed for the assessment of microbial community composition shifts in response to experimental treatments. Detailed methodologies regarding DNA extraction, primer design and validation, qPCR conditions, and data normalization procedures were based on previously established protocols [18]. DNA isolation was initiated by supplementing each sample with a lysis buffer (500 µL; composed of 100 mM NaCl, 50 mM Tris‐HCl, pH 8.0, and 100 mM EDTA), followed by the addition of 50 µL of 10% SDS, 20 µL of proteinase K, and 10 µL of lysozyme. The resulting mixtures were incubated at 56°C for 60 min to facilitate cell lysis and protein digestion. Mechanical disruption was then performed by introducing glass beads and vigorously vortexing the samples for 15 min. Purification of genomic DNA was achieved through a combined phenol‐chloroform extraction procedure and a commercial washing kit (GeneAll Co., Seoul, Korea). The integrity and purity of the isolated DNA were assessed using both NanoDrop spectrophotometry and agarose gel electrophoresis.

**Table 3:** Primer sequences used for PCR‐based detection of specific bacterial genera in the intestinal samples of juvenile Nile tilapia (*Oreochromis niloticus*).

Bacterium	Sequences		Amplicon size (bp)	Efficiency (%)	Reference
*Lactobacillus* sp.	F	TGGAAACAGRTGCTAATACCG	233	95.5	Byun et al. [38]
	R	GTCCATTGTGGAAGATTCCC			
*Vibrio* sp.	F	GGCGTAAAGCGCATGCAGGT	114	94.1	Tall et al. [39]
	R	GAAATTCTACCCCCCTCTACAG			
*Aeromonas* sp.	F	GAGAAGGTGACCACCAAGAACA	230	93.7	Yu et al. [40]
	R	CTGACATCGGCCTTGAACTC			
*Saccharomyces* sp.	F	GTGAAATTGTTGAAAGGGAA	260	97.5	Kesmen et al. [41]
	R	GACTCCTTGGTCCGTGTT			
*Bacillus* sp.	F	CGACGCTATTAACTATTACAACTGCTA	265	96.4	Haque et al., 2022 [42]
	R	GTAACAGCATGTGCCCTTGCA			
*Streptococcus* sp.	F	GTACAGTTGCTTCAGGACGTATC	197	97.8	Picard et al. [43]
	R	ACGTTCGATTTCATCACGTTG			
16S rRNA	F	ACTCCTACGGGAGGCAGCAG	200	98.2	Hoseini et al. [18]
	R	ATTACCGCGGCTGCTGG			

*Note*: The table lists target bacteria, primer sequences, expected amplicon sizes (bp), and literature references where available.

For subsequent PCR amplification, each 25 µL reaction contained 100 ng of purified DNA, 12.5 µL of RealQ Plus 2x Master Mix Green, and 0.25 µM of each gene‐specific primer. All amplifications were performed in triplicate to ensure reproducibility. Thermal cycling commenced with an initial denaturation step at 95°C for 15 min, followed by 40 cycles comprising denaturation at 94°C for 30 s, annealing at 56°C for 40 s, and extension at 72°C for 30 s. Melting curve analysis was conducted postamplification to verify primer specificity and amplicon homogeneity. Relative bacterial abundance was determined by normalizing target Ct values to both an endogenous reference (universal 16S rRNA gene) and CTL samples, with fold changes calculated using the ΔΔCt method.

### 2.6. Statistical Analysis

Statistical analysis was conducted to evaluate the effects of dietary treatments on the measured parameters. Before analysis, data were tested for normality and homogeneity of variances using the Shapiro–Wilk and Levene’s tests, respectively, to ensure that the assumptions of parametric tests were met. Subsequently, one‐way analysis of variance (ANOVA) was performed to determine significant differences among treatment groups, with significance set at *p* < 0.05. When ANOVA indicated significant effects, pairwise comparisons between treatment means were conducted using Tukey’s significant difference (HSD) post hoc test to CTL for type I error. All statistical analyses were carried out using GraphPad Prism version 10, while Levene’s test for the equality of variances was performed using IBM SPSS Statistics version 22. Data are presented as mean ± standard deviation unless otherwise specified.

## 3. Results

Growth performance, feed efficiency, and survival of juvenile Nile tilapia fed diets containing 0%, 10%, and 20% FSOC showed no significant differences among treatments (*p* > 0.05; Table 4). Initial weights were similar across all groups, confirming uniform starting conditions. Final weights, weight gain, and specific growth rates did not differ significantly, indicating that dietary inclusion of FSOC up to 20% had no adverse effect on growth. Feed conversion ratios also remained comparable among treatments, with no significant variation observed. Survival rates were 100% in all groups, demonstrating no negative impact of the diets on fish viability. These results suggest that FSOC can be incorporated into juvenile Nile tilapia diets at levels up to 20% without compromising growth performance, feed efficiency, or survival (Table 4).

**Table 4:** Growth performance, feed efficiency, and survival of juvenile Nile tilapia fed diets containing 0% (CTL), 10% (S‐10), and 20% (S‐20) fermented sesame oilcake.

Parameters	CTL	S‐10	S‐20	*p*‐Value
Initial weight (g)	13.37 ± 0.55	13.61 ± 1.01	13.37 ± 0.57	0.6098
Final weight (g)	41.91 ± 1.43	43.17 ± 1.24	40.47 ± 1.62	0.1498
Weight gain (%)	213.64 ± 5.67	217.93 ± 14.91	212.52 ± 10.74	0.8253
Specific growth rate (%/day)	2.04 ± 0.03	2.06 ± 0.08	2.03 ± 0.06	0.8352
Feed conversion ratio	1.20 ± 0.01	1.21 ± 0.01	1.22 ± 0.02	0.2490
Survival (%)	100	100	100	1.0000

*Note:* Values represent means ± standard deviation (*n* = 3).

The dietary inclusion of FSOC significantly influenced hepatic and intestinal antioxidant parameters in juvenile Nile tilapia (Table 5). In the liver, fish fed the 10% FSOC diet exhibited significantly higher SOD activity compared to the CTL and 20% inclusion groups (*p* < 0.05). Similarly, GSH levels were elevated in both 10% and 20% FSOC treatments relative to the CTL, with the 10% group showing the highest concentration (*p* < 0.05). MDA levels, an indicator of lipid peroxidation, were significantly reduced in fish receiving either level of FSOC compared to CTL (*p* < 0.05). Other liver antioxidant enzymes such as CAT, GPx, and Gr showed no significant differences among treatments (*p* > 0.05; Table 5).

**Table 5:** Hepatic and intestinal antioxidant parameters (mean ± SD; *n* = 3) of juvenile Nile tilapia fed diets containing 0% (CTL), 10% (S‐10), and 20% (S‐20) fermented sesame oilcake.

Parameters	CTL	S‐10	S‐20	*p*‐Value
Liver				
SOD (U/mg protein)	42.35 ± 3.82^b^	53.18 ± 3.63^a^	42.20 ± 4.58^b^	0.0245
CAT (U/mg protein)	66.70 ± 4.57	62.97 ± 7.21	70.07 ± 3.52	0.3325
GPx (U/mg protein)	63.32 ± 8.75	76.10 ± 9.48	66.15 ± 5.18	0.2035
Gr (U/mg protein)	40.70 ± 7.37	56.57 ± 8.11	50.50 ± 1.90	0.0601
GSH (μM/g wet weight)	1.82 ± 0.20^b^	3.04 ± 0.23^a^	2.57 ± 0.64^ab^	0.0281
MDA (nM/g wet weight)	60.63 ± 7.00^a^	36.63 ± 6.20^b^	40.60 ± 6.03^b^	0.0080
Intestine				
SOD (U/mg protein)	18.35 ± 2.53^b^	31.93 ± 4.17^a^	25.94 ± 4.07^ab^	0.0113
CAT (U/mg protein)	27.20 ± 4.33^b^	46.02 ± 8.19^a^	30.36 ± 3.66^b^	0.0148
GPx (U/mg protein)	29.37 ± 4.45^b^	43.73 ± 3.44^a^	38.36 ± 1.80^a^	0.0059
Gr (U/mg protein)	30.68 ± 3.44^b^	48.73 ± 4.87^a^	40.71 ± 6.64^ab^	0.0148
GSH (μM/g wet weight)	2.12 ± 0.29^b^	3.68 ± 0.43^a^	3.58 ± 0.72^a^	0.0175
MDA (nM/g wet weight)	40.12 ± 6.03^a^	26.10 ± 4.95^b^	25.97 ± 3.92^b^	0.0214

*Note*: Parameters measured include superoxide dismutase (SOD), catalase (CAT), glutathione peroxidase (GPx), glutathione reductase (Gr), reduced glutathione (GSH), and malondialdehyde (MDA). Different letters within each row indicate significant differences among treatments.

In the intestine, the 10% FSOC diet significantly enhanced activities of SOD, CAT, GPx, and Gr compared to CTL, with the 20% group showing intermediate or similar levels to the 10% group (*p* < 0.05; Table 5). Intestinal GSH levels were also significantly higher in both FSOC‐fed groups relative to the CTL (*p* < 0.05). Consistent with hepatic results, intestinal MDA concentrations were significantly lower in fish fed 10% and 20% FSOC compared to the CTL group (*p* < 0.05). These results indicate that moderate inclusion of FSOC in the diet improves antioxidant defenses and reduces oxidative stress markers in both liver and intestinal tissues, with the 10% inclusion level generally eliciting the most pronounced effects (Table 5).

The immunological parameters measured in the liver, plasma, skin mucus, and kidney of fish fed diets containing 10% and 20% FSOC showed significant modulation compared to the CTL diet (Table 6). In the liver, lysozyme activity and total Ig levels were significantly elevated in fish fed both inclusion levels of FSOC, with the highest values observed at 10% inclusion (*p* < 0.05; Table 6). Plasma lysozyme activity and total Ig concentration also increased significantly in the 10% FSOC group compared to the CTL, while AC activity showed no significant differences among treatments (*p* > 0.05; Table 6). In skin mucus, total Ig content was significantly higher in fish fed the 10% FSOC diet compared to CTL, whereas lysozyme, ALP, and protease activities showed no statistically significant changes (*p* > 0.05; Table 6). Kidney lysozyme activity was significantly enhanced in both FSOC groups relative to the CTL, while total Ig levels were not significantly affected (*p* > 0.05). Overall, dietary inclusion of FSOC at 10% notably enhanced several key humoral immune parameters across multiple tissues, indicating a positive immunostimulatory effect (Table 6).

**Table 6:** Immunological parameters in liver, plasma, skin mucus, and kidney of Nile tilapia fed diets containing 0% (CTL), 10% (S‐10), and 20% (S‐20) fermented sesame oilcake.

Parameters	CTL	S‐10	S‐20	*p*‐Value
Liver				
Lysozyme (U/mL)	33.70 ± 3.35^b^	49.57 ± 4.55^a^	46.77 ± 6.28^a^	0.0154
Total Ig (% of soluble protein)	15.77 ± 2.29^b^	31.40 ± 7.10^a^	27.40 ± 4.52^ab^	0.0215
Plasma				
Lysozyme (U/mL)	18.30 ± 3.26^b^	29.30 ± 4.23^a^	25.50 ± 4.57^ab^	0.0413
AC (U/mL)	101.40 ± 7.15	126.27 ± 15.79	114.73 ± 21.42	0.2390
Total Ig (g/L)	7.05 ± 0.38^b^	9.30 ± 1.03^a^	8.20 ± 0.72^ab^	0.0298
Skin mucus				
Lysozyme (U/mg protein)	23.92 ± 4.08	32.50 ± 2.69	28.71 ± 4.22	0.0790
Total Ig (% of soluble protein)	15.95 ± 2.79^b^	26.08 ± 4.75^a^	22.99 ± 3.01^ab^	0.0353
ALP (U/mg protein)	1.71 ± 0.13	2.76 ± 0.69	2.63 ± 0.45	0.0717
Protease (U/mg protein)	15.73 ± 2.17	15.73 ± 2.21	18.30 ± 1.88	0.2929
Kidney				
Lysozyme (U/mg protein)	30.82 ± 3.80^b^	44.79 ± 4.67^a^	48.48 ± 6.42^a^	0.0121
Total Ig (% of soluble protein)	20.04 ± 3.20	28.58 ± 3.69	25.50 ± 4.52	0.0858

*Note*: Data are presented as mean ± SD (*n* = 3). Parameters measured include lysozyme activity, total immunoglobulin (Ig) levels, alternative complement (AC) activity, alkaline phosphatase (ALP), and protease activity. Different letters within each row indicate significant differences among treatments.

**Figure 1:**
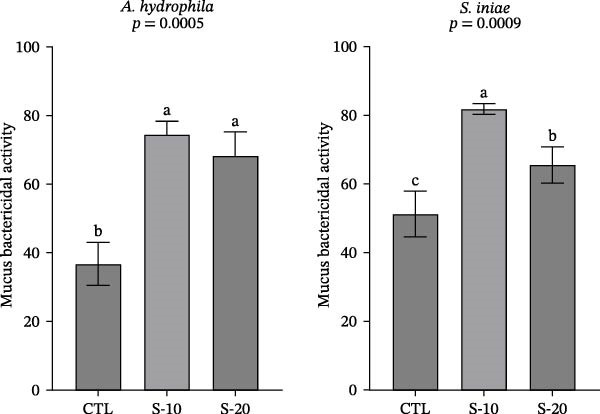
Skin mucus bactericidal activity (mean ± SD; *n* = 3) of fish fed diets containing 10% and 20% fermented sesame oilcake. Different letters above the bars indicate significant differences among treatments.

The skin mucus of fish fed diets containing FSOC (S‐10 and S‐20) showed significantly enhanced bactericidal activity against *A. hydrophila* and *S. iniae* compared to the CTL group (Figure 1; *p* < 0.05). Specifically, fish in the S‐10 and S‐20 groups exhibited higher inhibitory effects on *A. hydrophila*, with no significant difference between them, but both were significantly higher than the CTL. In contrast, bactericidal activity against *S. iniae* was highest in the S‐10 group, followed by the S‐20 group, both of which were significantly greater than the CTL (Figure 1; *p* < 0.05), and all treatments differed significantly from each other. These findings indicate that dietary inclusion of FSOC, particularly at 10%, enhances the nonspecific immune response of fish through improved skin mucus antimicrobial properties.

**Figure 2:**
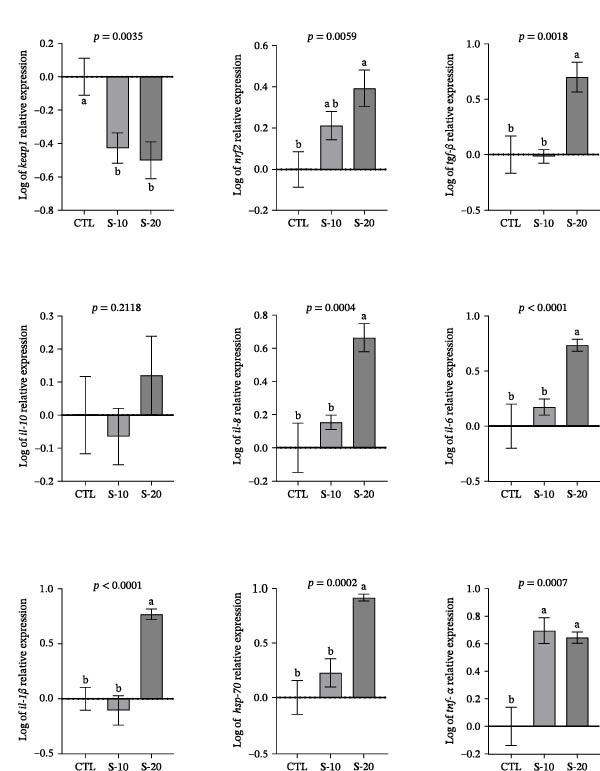
Gut transcript levels (mean ± SD; *n* = 3) of fish fed diets containing 10% and 20% fermented sesame oilcake. Different letters above/beneath the bars indicate significant differences among treatments.

The study examined the effects of diets containing 10% (S‐10) and 20% (S‐20) FSOC on the gut transcript levels of immune‐ and stress‐related genes in fish. Significant differences were observed in the expression of most genes. *keap1* relative expression was similar in S‐10 and S‐20 treatment, both significantly lower than that of the CTL group (Figure 2; *p* = 0.0035). *nrf2* relative expression showed a significant increase in the S‐20 group compared to the CTL and S‐10 (Figure 2; *p* = 0.0059). Similarly, *tgf-β* expression was significantly higher in both S‐10 and S‐20 groups than in CTL (*p* = 0.0018). In contrast, no significant changes were detected in *il-10* expression (*p* = 0.2118). For proinflammatory cytokines, *il-8*, *il-6*, and *il-1*β exhibited significantly elevated expression in the S‐10 and S‐20 groups compared to CTL (Figure 2; *p* = 0.0004, *p* < 0.0001, and *p* < 0.0001, respectively). The stress‐related gene *hsp-70* also showed higher expression in the fermented diet groups (*p* = 0.0002). Additionally, *tnf-α* expression was significantly upregulated in the S‐20 group compared to CTL and S‐10 (*p* = 0.0007). These results indicate that FSOC, particularly at the 20% inclusion level, enhances immune and stress responses in fish by modulating key gene pathways. The lack of effect on *il-10* suggests that its regulatory role may not be influenced by this dietary intervention. Overall, the findings highlight the potential of FSOC as a functional feed ingredient to improve fish health.

**Figure 3:**
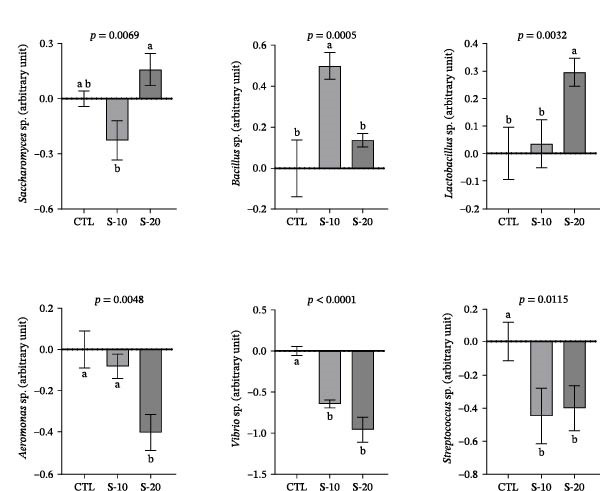
Gut microbial abundances (mean ± SD; *n* = 3) of fish fed diets containing 10% and 20% fermented sesame oilcake. Different letters above/beneath the bars indicate significant differences among treatments.

The gut microbial profiling revealed that dietary supplementation with FSOC significantly influenced the composition and abundance of key microbial taxa in the intestinal tract of fish. Among the assessed taxa, *Saccharomyces* sp. showed the most pronounced variation, with significantly lower abundance in the S‐10 group and the highest levels observed in the S‐20 group, suggesting a dose‐dependent enhancement of this yeast population (Figure 3). Similarly, *Bacillus* sp., known for their probiotic potential, were markedly more abundant in the S‐10 group compared to the CTL, indicating that moderate inclusion of FSOC may favor the proliferation of beneficial spore‐forming bacteria.

In contrast, the abundance of *Lactobacillus* sp., another probiotic genus, was the highest in the S‐20 group, suggesting that a higher inclusion level supports their enrichment more effectively. Opportunistic pathogens such as *Aeromonas* sp. and *Vibrio* sp. were significantly suppressed in both S‐10 and S‐20 groups relative to the CTL, indicating a potential antimicrobial effect of the FSOC against harmful intestinal microbes (Figure 3). Furthermore, *Streptococcus* sp. was more prevalent in the CTL group than in the supplemented groups, further supporting the notion that the dietary intervention may reduce the colonization of potentially pathogenic bacteria.

## 4. Discussion

The use of functional, upcycled feed additives is a key strategy for improving aquaculture sustainability. The present study demonstrates that incorporating FSOC into Nile tilapia diets maintains baseline growth while mechanistically enhancing systemic and mucosal defense pathways.

Specifically, our findings indicate that FSOC can be included in the diet of Nile tilapia at levels up to 20% without compromising growth performance or feed efficiency. A previous study similarly demonstrated that incorporating ~7%–15% FSOC (fermented by *Lactobacillus plantarum*) at the expense of a 5.4:1 wheat meal and fishmeal mixture exerted no detrimental effects on feed efficiency or nutrient digestibility [12]. In the present study, FSOC was substituted for a 1:1 mixture of soybean meal and poultry slaughterhouse by‐products. Despite this reduction in animal‐derived protein, the fish maintained normal growth, suggesting that FSOC is highly digestible in Nile tilapia and represents a highly viable, cost‐effective dietary ingredient when available. This outcome is particularly noteworthy given the higher crude fiber content in the S‐20 diet, which did not adversely affect growth performance or feed efficiency. This resilience may be explained by the distinct fiber profiles of soybean meal and sesame oilcake; soluble fiber constitutes approximately half of the total fiber in soybean meal, whereas it accounts for only about 20% in sesame oilcake. According to Amirkolaie et al. [44], insoluble fiber is well‐tolerated up to 8% of the diet without negative impacts on growth or fecal physical structure, unlike soluble fiber. Thus, the increased dietary fiber in the FSOC diets likely consisted of benign, insoluble fractions, explaining why growth and feed utilization remained unimpaired.

The elevated hepatic SOD activity and GSH levels, accompanied by a concurrent reduction in MDA content, indicate that dietary FSOC strengthens cellular redox balance at the superoxide level (the primary line of defense) and suppresses lipid peroxidation [45]. Bioactive compounds capable of scavenging ROS are also known to upregulate downstream antioxidant defense pathways [26]. These positive shifts can likely be attributed to yeast‐derived cellular components—such as nucleotides, beta‐glucans, and mannan oligosaccharides—which possess well‐documented antioxidant properties [46–48]. Comparable physiological benefits have been documented in Nile tilapia fed other yeast‐fermented feeds [49, 50].

Furthermore, the simultaneous upregulation of SOD, CAT, GPx, GR, and GSH, coupled with a significant decrease in intestinal MDA, suggests that FSOC induces a highly coordinated antioxidant response within the mucosal epithelium directly exposed to the lumen. As the primary site of exposure to feed‐derived bioactive elements, the intestine frequently exhibits activation of antioxidant defense mechanisms [51]. Previous research has shown that herbal ingredients and fermented fractions can enhance intestinal health and oxidative status, while enzymatic or probiotic supplements further bolster antioxidant defenses by improving digestibility and modulating the microbiota structure [52–54]. Additionally, sesame meal naturally contains lignans like sesamin and sesamolin, which are recognized for their potent antioxidant potential [55]. The fermentation process may have enhanced the bioavailability and bioactivity of these lignans, or yeast‐derived bioactive metabolites may have contributed to this protective mucosal barrier [56].

The innate (nonspecific) immune system serves as the primary defense barrier against invading pathogens, protecting fish prior to the maturation of slower, adaptive immune responses [57]. Consequently, the innate immune system is a major target for dietary interventions aimed at boosting disease resistance in aquaculture. Lysozyme is a key innate immune enzyme found across various fish tissues that targets Gram‐positive bacteria [58]. In this study, dietary FSOC significantly enhanced lysozyme activity in the plasma, liver, and kidney, alongside a positive trend toward increased activity in the skin mucus. These findings align with prior research demonstrating improved lysozyme activity in fish fed various fermented dietary components [59–61]. Although Igs are classical components of the adaptive immune system [62], dietary interventions have been shown to elevate baseline Ig levels in fish [63, 64]. This elevation in basal Igs, in tandem with other immunological enhancements, has been shown to boost host resistance against subsequent pathogen challenges [65, 66], pointing to an active role for basal Igs in fish disease resistance. Here, FSOC administration increased baseline Ig levels across multiple tissues, consistent with reports on other fermented feed ingredients in aquaculture diets [67–69]. While the precise pathways underlying this systemic upregulation of lysozyme and Ig levels remain to be fully elucidated, yeast‐derived components such as beta‐glucans and mannan oligosaccharides are known to stimulate these parameters upon dietary inclusion in fish [70, 71]. Furthermore, dietary administration of *Lactobacillus* sp. and *Bacillus* sp. has yielded similar immunomodulatory outcomes [72–75]. Thus, the enriched abundance of these beneficial taxa in the gut of FSOC‐treated fish may drive the observed enhancement of innate immunity.

As a dynamic primary barrier, fish skin mucus acts as a critical physical and biochemical shield against aquatic pathogens [76]. *A. hydrophila* and *S. iniae* represent two highly destructive pathogens in Nile tilapia aquaculture [77]. Our findings reveal that dietary FSOC significantly enhances the bactericidal capacity of skin mucus against both pathogens. This enhancement could lower the risk of aeromonad and streptococcal infections in farmed Nile tilapia, particularly since skin mucus represents the first line of defense against waterborne pathogens [76]. In support of these findings, prior studies have documented improved mucosal bactericidal activity following the administration of fermented feedstuffs [78, 79]. While the underlying mechanisms warrant further investigation to be fully defined, yeast cell‐wall components and the enrichment of gut *Bacillus* sp. and *Lactobacillus* sp. are highly plausible contributors to this enhanced mucosal defense [75, 80–82].

The transcription factors *nrf2* and *keap1* play pivotal roles in managing cellular oxidative stress by activating downstream antioxidant genes [83]. Given the observed improvements in antioxidant enzyme activities, GSH levels, and MDA suppression in the intestine, it is highly likely that the enhanced mucosal antioxidant capacity in FSOC‐treated fish was mediated through the activation of the *nrf2*/*keap1* signaling pathway. Similar modulations of molecular and biochemical antioxidant pathways have been observed with other fermented dietary ingredients [84, 85]. Concurrently, *hsp-70* acts as a molecular chaperone, preventing protein misfolding and denaturation during oxidative and cellular stress [86]; its upregulation here may stem from yeast‐derived components in the FSOC, as supported by previous findings [87]. Collectively, these results suggest that FSOC enhances intestinal cellular resilience through a bidirectional mechanism involving both the *nrf2*/*keap1* signaling axis and chaperone‐mediated protective pathways.

At first glance, upregulation of proinflammatory cytokines such as *il-1*β, *il-6*, *il-8*, and *tnf-α* in the intestinal mucosa might suggest localized inflammation [88]. However, given the concurrent suppression of oxidative stress and the lack of negative impacts on growth performance, this upregulation is more accurately characterized as beneficial immunostimulation. Furthermore, *tgf-β* and *il-10* are critical regulatory cytokines that prevent runaway immune responses, foster mucosal tolerance, and initiate tissue repair [89]. In our study, although proinflammatory cytokine expression was elevated in the S‐20 group, intestinal *tgf-β* expression was significantly upregulated, and *il-10* expression showed an increasing trend. This balanced expression profile indicates that FSOC does not provoke unregulated intestinal inflammation but instead primes the mucosal immune system into a regulated state of high alertness. In other words, FSOC low‐levelly stimulates mucosal immune cells, enhancing their readiness to respond to potential pathogen challenges. By maintaining this preactivated immunological state, the host can mount a more rapid and robust defense upon pathogen exposure.

FSOC clearly exerts a profound modulatory effect on the intestinal microbial landscape. We observed a significant enrichment of potentially beneficial microorganisms, alongside a corresponding decrease in opportunistic and pathogenic taxa in FSOC‐treated fish. This taxonomic shift suggests that FSOC optimizes the probiotic‐to‐pathogen ratio within the intestinal ecosystem, thereby bolstering gut health and barrier integrity. While the exact mechanisms driving these microbial shifts require further study, yeast biomass present within FSOC likely played a key role; indeed, dietary supplementation with *S. cerevisiae* has been shown to induce comparable taxonomic shifts in the gut microbiota of largemouth bass, *Micropterus salmoides* [90]. Furthermore, prebiotic components such as beta‐glucans and mannan oligosaccharides are well known to modulate gut microbial profiles when used as feed additives [91, 92], which further explain the microbial dynamics observed in the present study.

Beneficial taxa such as *Saccharomyces*, *Bacillus*, and *Lactobacillus* species act as probiotics within the fish gastrointestinal tract, stimulating host immunity and maintaining epithelial barrier integrity [93–95]. These microorganisms also suppress pathogens by producing short‐chain fatty acids, organic acids, and various antimicrobial compounds [96, 97]. The reduction of opportunistic pathogens observed in this study suggests that FSOC shapes the gut microbiome into a more protective state. This protective shift likely operates via two primary pathways: (1) competitive exclusion, where increased beneficial populations limit colonization niches and suppress the proliferation of pathogens, and (2) the direct inhibitory action of fermentation‐derived antimicrobial agents, such as organic acids, peptides, and phenolic derivatives produced during FSOC processing. These favorable microbial transitions are highly consistent with the observed intestinal gene expression profiles and mucosal immune enhancements. Beneficial microbes provide immunological priming through Toll‐like receptor signaling pathways on mucosal immune cells, while the reduced pathogen burden simultaneously mitigates local oxidative stress and inflammatory pressure [97, 98]. Further studies simultaneously investigating genomic responses and whole microbiome analysis (such as RNA‐seq and next‐generation sequencing) are encouraged for robust addressing of this topic.

## 5. Conclusion

In conclusion, this study demonstrates that FSOC can be effectively incorporated into Nile tilapia diets at up to 20% inclusion, maintaining optimal growth while significantly enhancing host defense mechanisms. The dietary administration of FSOC bolsters systemic and mucosal immunity, strengthens antioxidant capacity via the *nrf2*/*keap1* signaling pathway, and promotes a beneficial shift in the intestinal microbiota. By priming both the skin and intestinal barriers, FSOC increases the physiological readiness of the fish against potential pathogens. These results highlight FSOC as a valuable, sustainable feed ingredient that integrates nutritional efficiency with robust health‐promoting properties. Ultimately, utilizing FSOC offers a practical and eco‐friendly strategy to improve the overall resilience and productivity of Nile tilapia aquaculture. Further studies are encouraged to assess the effects of longer period of fermentation of sesame oilcake with *S. cerevisiae* for potential improvement of nutritional profile. By this, positive CTL diets including non‐FSOC should be included in the study designs to illustrate the biological benefits of FSOC in Nile tilapia.

## Author Contributions

**Seyyed Morteza Hoseini:** conceptualization, project administration, writing – draft, editing final version. **Morteza Yousefi:** conceptualization, writing – draft. **Alireza Afzali-Kordmahalleh:** methodology, investigation, formal analysis, writing – draft. **Seyed Hossein Hoseinifar:** investigation, writing – draft. **Hamed Abdollahpour and Mert Minaz:** writing – draft, editing final version.

## Funding

This work was supported by the RUDN University Scientific Projects Grant System under Project Number 040728‐2‐000.

## Ethics Statement

This study was conducted following the Institutional Animal Care and Use Committee (IACUC) guidelines of the University of Tehran under Approval Code IR.UT.VETMED.REC.1403.063.

## Conflicts of Interest

The authors declare no conflicts of interest.

## Data Availability

Data used in this study are available upon reasonable request from the corresponding authors.
